# Hydrolyzed conchiolin protein inhibits melanogenesis through PKA/CREB and MEK/ERK signalling pathways

**DOI:** 10.1111/ics.13012

**Published:** 2024-08-11

**Authors:** Yaqi Zhang, Sisi Wang, Anquan Yang

**Affiliations:** ^1^ Osmum Biological Co., Ltd Huzhou China

**Keywords:** hydrolyzed conchiolin protein, MEK/ERK signalling pathways, melanogenesis, PKA/CREB signalling pathway

## Abstract

**Objective:**

Hydrolyzed conchiolin protein (HCP) derived from pearl and nacre extracts exerts skin‐lightening effects; however, the underlying molecular mechanisms are not fully understood. Herein, we investigated the effect of HCP on melanogenesis and the signalling pathways involved.

**Methods:**

B16F10 cells and PIG cells were treated with HCP to verify its ability to inhibit melanin. Western Blot, immunofluorescence, and flow cytometry methods were performed to investigate the effect of HCP on melanogenesis signalling pathway proteins. The inhibitors were used to further validate the effect of HCP on PKA/CREB and MEK/ERK signalling pathways. To further evaluate the whitening ability of HCP, changes in melanin were detected using 3D melanin skin model and zebrafish model.

**Results:**

HCP was found to significantly inhibit melanin synthesis and decrease the expression of melanogenesis‐related proteins, such as microphthalmia‐associated transcription factor (MITF), tyrosinase, and tyrosinase‐related protein‐2, in a dose‐dependent manner. Additionally, we revealed that HCP suppresses melanogenesis via the regulation of the PKA/cAMP response element‐binding (CREB) and MEK/extracellular signalling‐regulated kinase (ERK) signalling pathways. Using 3D melanin skin models, we demonstrated that HCP can achieve skin‐lightening effects by improving apparent chroma, increasing apparent brightness, and inhibiting melanin synthesis. Furthermore, HCP exhibits skin‐whitening effects in a zebrafish model.

**Conclusion:**

These results suggest that HCP suppresses the melanogenesis signalling cascade by inhibiting the PKA/CREB, MEK/ERK signalling pathway and downregulating MITF and its downstream signalling pathways, resulting in decreased melanin synthesis. In summary, HCP is a potential anti‐pigmentation agent with promising applications in cosmetics and pharmaceutical products.

## Graphical abstract

Our findings suggest that Hydrolyzed conchiolin protein (HCP) possesses an anti‐melanogenic properties. HCP affects MITF expression and inhibits melanogenesis by regulating the PKA/CREB, MEK/ERK pathways.

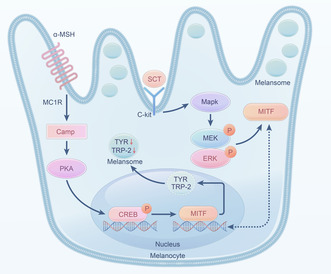

## Résumé

**Objectif:**

La protéine conchioline hydrolysée (HCP) dérivée des extraits de perle et de nacre exerce des effets éclaircissants sur la peau ; cependant, les mécanismes moléculaires sous‐jacents ne sont pas entièrement compris. Dans cette étude, nous avons investigué l'effet de la HCP sur la mélanogenèse et les voies de signalisation impliquées.

**Méthodes:**

Les cellules B16F10 et PIG ont été traitées avec la HCP pour vérifier sa capacité à inhiber la mélanine. Des méthodes de Western Blot, d'immunofluorescence et de cytométrie en flux ont été réalisées pour étudier l'effet de la HCP sur les protéines des voies de signalisation de la mélanogenèse. Les inhibiteurs ont été utilisés pour valider davantage l'effet de la HCP sur les voies de signalisation PKA/CREB et MEK/ERK. Pour évaluer plus en détail la capacité éclaircissante de la HCP, les changements de mélanine ont été détectés en utilisant un modèle de peau en 3D de mélanine et un modèle de poisson‐zèbre.

**Résultats:**

Il a été constaté que la HCP inhibe significativement la synthèse de la mélanine et diminue l'expression des protéines liées à la mélanogenèse, telles que le facteur de transcription associé à la microphthalmie (MITF), la tyrosinase et la protéine liée à la tyrosinase‐2, de manière dose‐dépendante. De plus, nous avons révélé que la HCP supprime la mélanogenèse via la régulation des voies de signalisation PKA/cAMP et MEK/ERK. En utilisant des modèles de peau en 3D de mélanine, nous avons démontré que la HCP peut atteindre des effets éclaircissants sur la peau en améliorant la chroma apparente, en augmentant la luminosité apparente et en inhibant la synthèse de la mélanine. En outre, la HCP présente des effets éclaircissants sur la peau dans un modèle de poisson‐zèbre.

**Conclusion:**

Ces résultats suggèrent que la HCP supprime la cascade de signalisation de la mélanogenèse en inhibant les voies de signalisation PKA/CREB et MEK/ERK et en régulant à la baisse le MITF et ses voies de signalisation en aval, ce qui entraîne une diminution de la synthèse de la mélanine. En résumé, la HCP est un agent potentiel anti‐pigmentation avec des applications prometteuses dans les produits cosmétiques et pharmaceutiques.

## INTRODUCTION

Melanin is a pigment that is widely present in human skin, hair, iris, and other tissues. This pigment serves to resist ultraviolet radiation, protect DNA, and prevent skin burns and cancer [1]. However, excessive melanin production can cause pigmentary skin disorders such as melasma, freckles, age spots, and hyperpigmentation [2, 3]. The process of melanogenesis is increasingly well understood; tyrosinase (TYR), a key enzyme in this process, promotes melanogenesis together with tyrosinase‐related protein‐1 (TRP‐1) and tyrosinase‐related protein‐2 (TRP‐2) [4]. Microphthalmia‐associated transcription factor (MITF) participates in regulating the proliferation, survival, and differentiation of melanocytes as well as controls TYR, TRP‐1, and TRP‐2 expression. This transcription factor is a master regulator of melanin synthesis [5, 6]. The three most common signalling pathways are involved in regulating melanogenesis: the protein kinase A (PKA)/cAMP response element‐binding protein (CREB), Wnt/β‐catenin, and mitogen‐activated protein kinase (MAPK) pathways. MITF serves as a central hub protein in these three major pathways. Under ultraviolet radiation, melanocytes produce α‐melanocyte‐stimulating hormone (α‐MSH) and endothelin‐1 (ET‐1) [7, 8]. α‐MSH increases intracellular cyclic adenosine monophosphate (cAMP) levels and activates the PKA/CREB pathway when bound to melanocortin kinin 1 receptor (MC1R) on the membrane of melanocytes, enhancing MITF expression and ultimately promoting melanin production. [9, 10, 11]. Another signalling pathway that targets *MITF* gene expression is the Wnt/β‐catenin signalling pathway. Activation of the Wnt pathway inhibits phosphorylation of glycogen synthase kinase 3β (GSk3β), causing the accumulation of β‐catenin [12]. Accumulated β‐catenin is transported to the nucleus, where it forms a complex with T cell factor/lymphoid‐enhancing factor‐1 (TCF/LEF), yielding in increased MITF expression [13, 14, 15]. Therefore, GSk3β can be activated to inhibit melanin synthesis through the Wnt pathway. Additionally, the phosphorylation of MAPKs, including extracellular signal‐regulated kinases (MEK/ERK), c‐Jun N‐terminal kinase (JNK), and p38, effectively regulates MITF transcription and promotes melanin synthesis [16, 17, 18].

Pearls are ancient organic gemstones cherished by people worldwide. In China, the medicinal use of pearl and nacre has a historyk of over 2000 years. As documented in the Ming Dynasty's Compendium of Materia Medica: ‘Applying pearl powder to the face can make the skin moist and improve complexion.’ Pearl and nacre are considered to possess excellent skin‐whitening effects. In recent years, there have been numerous studies worldwide on the composition of the skin‐whitening ingredients present in pearl and nacre, and the underlying mechanism. We have also previously found that pearl extract has a melanin‐inhibiting effect on mouse melanoma cells [19]. However, the mechanism of HCP's whitening effect is not well understood, and there are not many studies on this topic. Shan Yang investigated the mechanism of whitening effect of hydrolyzed conchiolin protein (HCP), derived from pearl powder, on melanogenesis in MNT‐1 human melanoma cells [20]. HCP can promote the transfer of melanosomes from MNT‐1 melanocytes to HaCaT cells, thereby accelerating the skin whitening process through rapid transfer and metabolism of melanosomes during keratinocyte differentiation. The paper mainly explores the impact of HCP on melanosome transfer. In our study, we are more inclined to explore the impact of HCP on the melanin production signalling pathway. HCP from freshwater pearl mussel (*Hyriopsis cumingii*) shells was found to inhibit melanogenesis via the PKA/CREB and MEK/ERK signalling pathways in B16F10 cells and PIG1 cells, thus validating its skin‐whitening effects. The anti‐melanogenic effect of HCP was further confirmed based on human 3D skin and zebrafish models.

## MATERIALS AND METHODS

### HCP preparation

*Hyriopsis cumingii* was purchased from Dongying Jewellery Co., Ltd. (Zhuji City, China). The shells were washed with clean water, crushed using an industrial grinder PG‐85 and sieved to obtain shell powder with particle sizes ranging from 20 to 100 mesh (840–149 μm sieve aperture). The shell powder was mixed with a 20% acetic acid solution, which was 10 times the weight of the shell powder. The mixture was stirred continuously at 60 rpm until it changed from white to pale yellow, and no bubbles were generated, indicating the completion of the reaction. The completely reacted mixture was centrifuged at 8000–12000 rpm (Tubular centrifuge GQ150, China), and the supernatant was discarded to obtain the conchiolin protein precipitate. The conchiolin protein precipitate was continuously washed with deionized water on a 100 mesh (149 μm sieve aperture) gauze until the TDS value (total dissolved solids) of the washing liquid was ≤100 ppm, indicating that the conchiolin protein was washed clean. The washed conchiolin protein was dried and its nitrogen content was measured using a Kjeldahl nitrogen analyser (Haineng K1100, China), which was found to be between 16%–16.64%. The washed dry weight of 1 kg of conchiolin protein was mixed thoroughly with 20 kilograms (kg) of deionized water and 0.2 kg of papain (6000 U/mg).The mixture was enzymatically digested continuously at pH 6.0–6.5, 60°C, and 200 rpm for 5 h. After enzymatic digestion, the digestion mixture was centrifuged at 8000–12000 rpm for 5–10 min, and the supernatant was collected as the enzymatic digestion solution. The enzymatic digestion solution was filtered using a 200 nm ceramic membrane and then subjected to membrane separation using a 1000 Da ultrafiltration membrane to remove enzymes and other large molecules. The separated solution was concentrated using a 200 Da nanofiltration membrane. The obtained concentrated solution was freeze‐dried (GZL‐5 vacuum freeze dryer) to obtain HCP freeze‐dried powder. The yield of HCP freeze‐dried powder was between 0.38%–0.4% (based on the weight of the shell powder). The peptide content of the HCP freeze‐dried powder was determined using the dinitrophenylhydrazine method [21], and it was found to be between 38%–46%.

### Cell culture

B16F10 mouse melanoma cells were obtained from the American Type Culture Collection (ATCC, CRL 6475, USA). PIG1 cells were purchased from BLUEFBIO (BFB, BFN60808828, China). B16F10 and PIG1 cells were cultured in Dulbecco's Modified Eagle's Medium (Sigma‐Aldrich, USA), with 10% fetal bovine serum (Hyclone, USA), and 1% mycillin (Sigma‐Aldrich). Cells were maintained in a 37°C incubator containing 5% carbon dioxide.

### Cell viability assay

The viability of B16F10 cells and PIG1 cells were measured using a CCK‐8 solution (Beyotime, China) assay. Cells were seeded at a density of 2 × 10^5^ B16F10 cells or 1 × 10^5^ PIG1 cells per well in 96‐well plates and incubated for 24 h. The experimental group was supplemented with different concentrations of HCP. In the control group, medium was replaced only with new medium and incubated for 24 h. The culture medium was discarded and replaced with serum‐free basal culture medium. Then, 10 μL of CCK‐8 solution was added to each well, followed by incubation at 37°C for 1–2 h. Absorbance was detected at 450 nm with a Multiscan spectrum microplate reader (Thermo, USA) [22]. All tests were repeated three times.

### Measurement of cellular melanin content

B16F10 cells were seeded at a density of 6 × 10^5^ cells per well in 10‐cm dishes and incubated for 24 h. The experimental group was supplemented with 12.5 μg/mL, 25 μg/mL and 50 μg/mL HCP, whereas in the control group, the medium was replaced with new medium and incubated for 24 h. PIG1 cells were seeded at a density of 4 × 10^5^ cells per well in 10‐cm dishes and incubated for 24 h. They were then treated with 10 μg/mL, 20 μg/mL and 50 μg/mL HCP in the presence or absence of 100 nM α‐MSH for 24 h. The culture medium was discarded and washed twice with PBS. B16F10 and PIG1 cells were then collected through trypsinization, and the supernatant was discarded and washed twice with PBS. Cells were lysed by adding 300 μL of 1 N NaOH solution containing 10% dimethyl sulfoxide (DMSO) to each tube and placed in a water bath at 80°C for 30 min. Next, 100 μL of cell lysate was pipetted into a 96‐well plate and absorbance at 475 nm was measured with a Multiscan spectrum microplate reader (Thermo) [23, 24]. All tests were repeated three times.

### Western blotting

B16F10 cells were seeded at a density of 4 × 10^5^ cells per well in 10‐cm dishes and incubated for 24 h. The experimental group was supplemented with HCP at different concentrations, while in the control group, medium was replaced with a new medium only. The protein was collected after 24 h of incubation, and the total protein was extracted using a protein extraction kit (Beyotime, China). Protein concentration was determined using the Total Protein Extraction Kit (Takara, Japan), followed by separation on a 10% sodium dodecyl sulfate‐polyacrylamide gel electrophoresis (SDS‐PAGE) gel (GenScript, China). The proteins were then transferred onto a polyvinylidene‐fluoride (PVDF) membrane (Millipore, USA) under a current of 200 mA for 90 min. The membrane was then removed, blocked with 5% skimmed milk for 1 h, and incubated with the primary antibody overnight at 4°C, followed by incubation with the secondary antibody for 1 h at room temperature. The chemiluminescence signals were captured using a chemiluminescence imaging system (Protein Simple, USA), and the grayscale ratio of the target protein to the corresponding internal reference protein was analysed with Image J software (National Institute of Health, Bethesda, MA, USA). The experiment was independently repeated three times. The primary antibodies TYR (dilution, 1:10000; ab170905, UK), TRP‐2 (dilution, 1:3000; ab221144), ERK (dilution, 1:10000; ab184699), p‐ERK (dilution, 1:2000; ab76299), PKA (dilution, 1:1000; ab75991), MITF (dilution, 1:3000; CST‐12590, USA), MEK (dilution, 1:5000; CST‐9122), p‐MEK (dilution, 1:3000; CST‐9121), CREB (dilution, 1:4000; CST‐9197), and p‐CREB (dilution, 1:3000; CST‐9198) were used in the experiments; antibodies selected for internal reference proteins were β‐actin (dilution, 1:8000; CST‐4967), as well as secondary rabbit and mouse antibodies (Abmart, China) with horseradish peroxidase (HRP)‐conjugated.

### Immunofluorescence (IF) detection

PIG1 cells were seeded at a density of 5 × 10^5^ cells per well in six‐well plates and incubated for 24 h. The cells were divided into three groups: the blank control group did not receive any treatment, the model group received 100 nM α‐MSH (MCE, USA) without HCP, and the experimental group received 100 nM α‐MSH and 50 μg/mL HCP. When the cell density was microscopically observed to be approximately 60%, each group was subjected to the corresponding treatments, followed by incubation for 24 h. Supernatants from the six‐well plates were aspirated and discarded, and cells were washed three times with PBS for 5 min each time. Next, 500 μL 4% paraformaldehyde was added for fixation for 15 min. The paraformaldehyde fixative was then discarded; this was followed by washing with PBS 3 times for 5 min each time; addition of 500 μL 0.1% TritonX‐100, incubation at room temperature for 10 min, and another washing step with PBS, for three times, 5 min each time. Then, incubation was performed with blocking solution at room temperature for 30 min. The blocking solution was discarded; primary antibody was next added and incubated overnight at 4°C. Subsequently, another wash step with PBS was performed, three times, for 5 min each time. Secondary antibody was then added, followed by incubation in the dark for 1 h. Another wash with PBS was performed three times, for 5 min each time. Then, DAPI was added for staining, followed by incubation in the dark for 5 min and washing with PBS three times, 5 min each time. Then, one drop of anti‐fluorescence quenching mounting solution was dropped on to a slide; the coverslip was covered with cells, avoiding bubbles, and the cells were allowed to contact the mounting solution. Observations were performed using a confocal laser scanning microscope (Zeiss LSM980, Germany Zeiss) with randomly selected fields for photography.

### Flow cytometry

PIG1 cells were seeded at a density of 5 × 10^5^ cells/well in six‐well plates and incubated for 24 h. The old medium was discarded and cells were washed twice with 2 mL PBS. Then, cells were collected by trypsinization, and the supernatant was discarded. Subsequently, 1 mL of 3% bovine serum albumin (BSA) was gently added and pipetted to resuspend the cells. The cells were centrifuged at 800 rpm for 5 min, and the supernatant was aspirated. The washing process with 3% BSA was repeated twice. Fixation/Permeabilization Concentrate (4×) was diluted with fixation/permeabilization diluent to prepare a 1× solution. Then, 1 mL of fixation/permeabilization concentrate (1×) was added per tube, and incubation was carried out at 4°C for 40 min. Permeabilization buffer (10×) was diluted with ultrapure water to prepare a 1× solution. The cells were washed twice with permeabilization buffer (1×), centrifuged at 800 rpm for 5 min, and the supernatant was discarded. To each tube, 100 μL of MITF Antibody (diluted 1:50; Proteintech, USA) and IgG (diluted 1:50; Bioss, China); or 100 μL of TYR Antibody (diluted 1:30; Abcam) and IgG (diluted 1:30; Bioss); or an equivalent amount of buffer, was added. Incubation was carried out at 4°C for 40 min. The cells were washed twice with permeabilization buffer (1×), centrifuged at 800 rpm for 5 min, and the supernatant was discarded. Then, to each tube, we added 100 μL of the fluorescent secondary antibody (CoraLite488‐conjugated Goat Anti‐Rabbit IgG (H + L), diluted 1:1000; Proteintech), and incubation was performed in the dark at room temperature for 1 h. The cells were then washed twice with permeabilization buffer (1×), centrifuged at 800 rpm for 5 min, and the supernatant was discarded. The cells were then resuspended in the permeabilization buffer (1×) and transferred to the flow cytometer for analysis.

### Evaluation and determination of the inhibitory effect of melanin in zebrafish embryos

Zebrafish embryos at 6 h post fertilization (hpf) with normal development were selected and placed in a 12‐well plate containing embryo culture medium, with five embryos per well. Each well was then filled with 2 mL of the corresponding concentration of HCP solution and arbutin solution. The culture plate was covered and wrapped with aluminium foil, and incubation was carried out in a light‐avoiding biochemical incubator at 28.5 ± 1°C. After incubation for 45 hpf, the melanin signal intensity (S) in the heads of the zebrafish was analysed using Image J and photographed microscopically with an SZX7 dissecting microscope (OLYMPUS, Japan). The inhibitory effect of HCP on melanin was calculated using the formula: C = 100% × (S_0_ − S_1_)/S_0_, where C represents the melanin inhibition rate, S_0_ is the average melanin signal intensity in the blank control group, and S_1_ is the average melanin signal intensity in the test group.

### Evaluation and determination of the whitening effect of 3D reconstructed human melanin epidermal model

The whitening efficacy of HCP was assessed by testing the 3D melanin skin model (MelaKutis®) for changes in apparent chroma, apparent brightness (L*value), melanin content, and melanin distribution of the model after treatment. The test was performed by Shanxi BioCell General Testing Co., Ltd. The experiment comprised a blank control group, negative control group, positive control group, and sample group (Table 1 for specific protocol). Stimulation with 5 nM ET‐1 was performed daily. The positive control kojic acid and configured HCP were evenly spread on the melanin model surface by surface administration after three consecutive days of stimulation (each dose, 10 μL) every 2 days for four consecutive days. At the end of model culture, photographs were taken using a camera for photography of changes in apparent chroma, L* value detection, and melanin content detection. Three models from the experimental group were fixed in 4% paraformaldehyde solution for 24 h. After embedding and sectioning, staining was performed in accordance with the instructions of Masson‐Fontana melanin stain kit (Bioss, China), and photographs were taken under a microscope.

**TABLE 1:** Information on experimental groups of the 3D melanin skin model.

Group	Sample name	Dose concentration	Stimulation condition	Test model	Test indicators	Test method
Blank Control (BC)	/	/	/	MelaKutis®	1. Apparent chroma 2. L* value 3. Melanin content 4. Melanin distribution	1. Photography 2. Chroma meter test 3. Alkaline lysis 4. Silver staining
Negative Control (NC)	/	/	ET‐1			
Positive Control (PC)	Kojic acid	500 μg/mL				
Sample group	HCP	0.1%, 0.01%, 0.001% (m/v)				

## RESULTS

### Effect of HCP on the viability of B16F10 cells and PIG1 cells, and melanogenesis‐inhibiting effects

**FIGURE 1:**
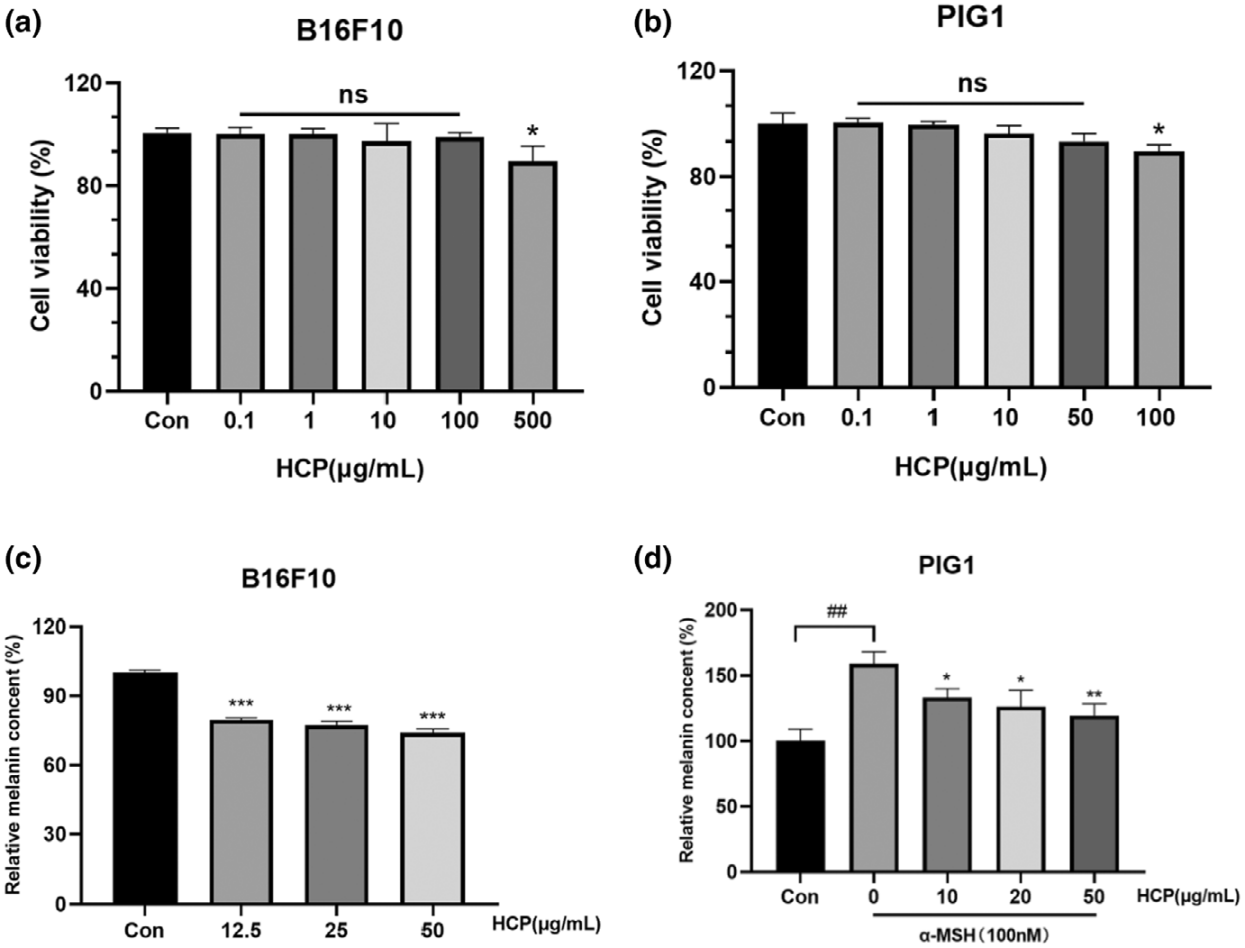
(a, b) Effect of HCP on the viability of B16F10 and PIG1 cells. B16F10 and PIG1 cells were subjected to treatment with HCP at several concentrations for a duration of 24 h, and the CCK8 assay was used to measure cell viability. (c, d) Effect of HCP on melanin synthesis in B16F10 and PIG1 cells. Percentages in treatment groups were calculated relative to those in the control (Con) group. Data are displayed in the form of mean ± SD (*n* = 3). *p<0.05, **p < 0.01, ***p < 0.001 compared with the control group.

The non‐toxic concentration range of HCP for B16F10 cells and PIG1 cells was determined by the CCK8 method. The results showed that HCP was non‐toxic to B16F10 and PIG1 cells in the range of 0–100 μg/mL (Figure 1a) and 0–50 μg/mL (Figure 1b), respectively. Additionally, we evaluated the anti‐melanogenic effects of HCP on B16F10 and PIG1 cells. The results show that at concentrations of 50 μg/mL, 25 μg/mL, and 12.5 μg/mL, HCP significantly reduced the melanin content in B16F10 cells by 26.1%, 22.6%, and 20.4%, respectively (Figure 1c). In PIG1 cells, HCP inhibited α‐MSH‐induced melanin production at concentrations of 50 μg/mL, 20 μg/mL, and 10 μg/mL, resulting in reductions of 39.5%, 32.7%, and 25.8%, respectively (Figure 1d).

### HCP inhibits PKA/CREB and MEK/ERK pathway protein expression in B16F10 cells

**FIGURE 2:**
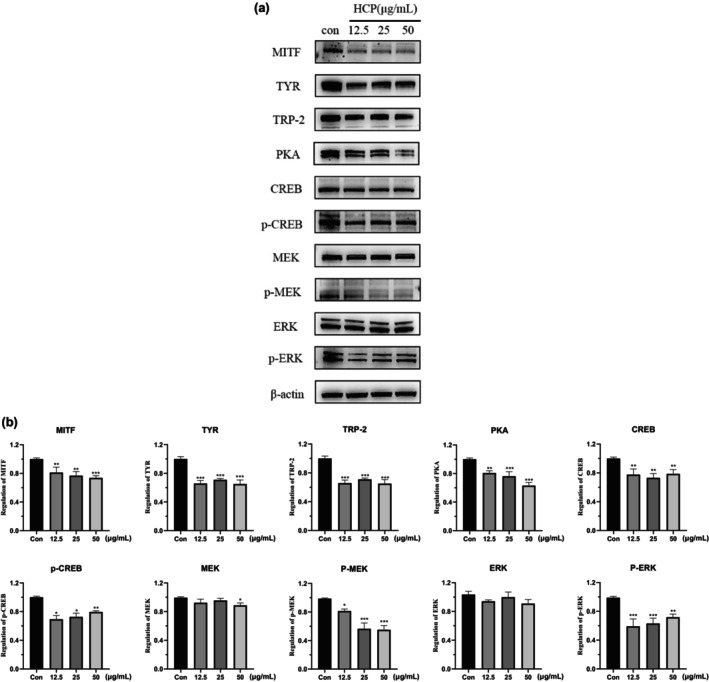
Effect of HCP on the expression levels of melanogenesis‐related proteins in B16F10 cells. B16F10 cells were subjected to treatment with 12.5, 25, 50 μg/mL HCP for a duration of 24 h. The levels of the TYR, TRP‐1, MITF, PKA, ERK, and p‐ERK proteins were analysed by western blotting. Equal protein loading was confirmed through a comparison with β‐Actin expression. Data are displayed in the form of mean ± SD (*n* = 3). P value compared with the control (Con) group. *p < 0.05, **p < 0.01, ***p < 0.001.

To investigate whether the anti‐melanogenic activity of HCP is correlated with changes in the expression levels of melanogenesis‐related proteins, including TYR, TRP‐1, MITF, PKA, CREB, p‐CREB, MEK, p‐MEK, ERK, and p‐ERK, cells were exposed to HCP for 24 h. Then, western blotting was performed on the extracts. The expression of the proteins was found to be suppressed by HCP treatment (Figure 2a,b). These findings suggest that the inhibition of melanogenesis by HCP is related to the downregulation of the PKA/CREB or MEK/ERK signalling pathway.

### HCP inhibits α‐MSH‐induced MITF and TYR protein expression in PIG1 cells

**FIGURE 3:**
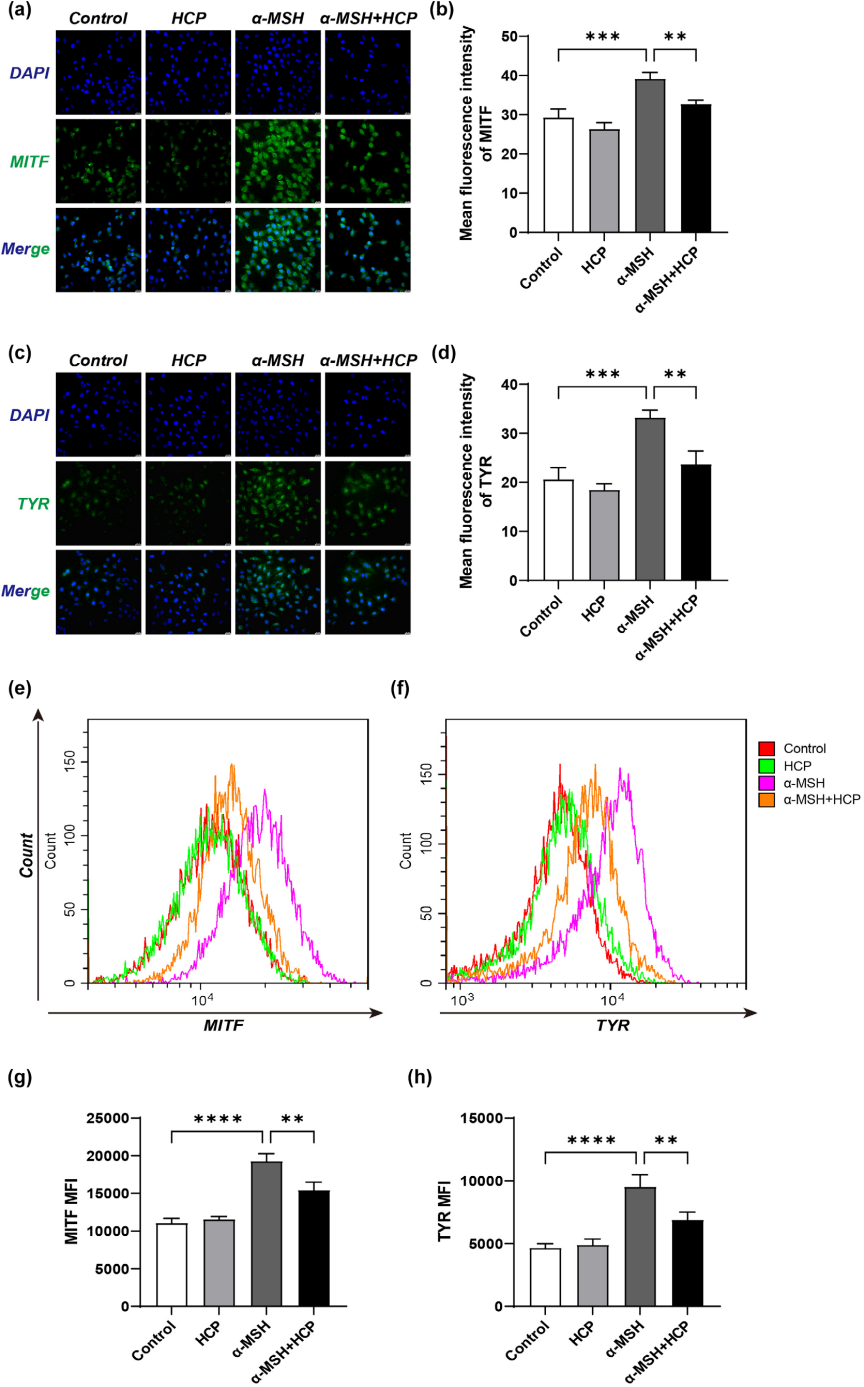
Effect of HCP on the expression levels of MITF and TYR proteins in PIG1 cells. PIG1 cells were subjected to treatment with 50 μg/mL HCP with (or without) 100 nM α‐MSH for 24 h. The MITF and TYR proteins levels were analysed by Iimmunofluorescence (a, b, c, d) and FC (e, f, g, h). Data are displayed as mean ± SD (*n* = 3). *p < 0.05, **p < 0.01, ***p < 0.001.

IF and flow cytometry (FC) were used to examine the effect of HCP on MITF and TYR protein expression in PIG1 cells stimulated with α‐MSH. As shown in Figure 3, the expression levels of MITF and TYR in PIG1 cells significantly increased upon induction with α‐MSH. However, compared with the α‐MSH group, the expression levels of MITF and TYR in the α‐MSH + HCP group were significantly decreased. These findings indicate that HCP can inhibit MITF and TYR protein expression in PIG1 cells stimulated by α‐MSH.

### HCP inhibits melanogenesis through the PKA/CREB pathway

**FIGURE 4:**
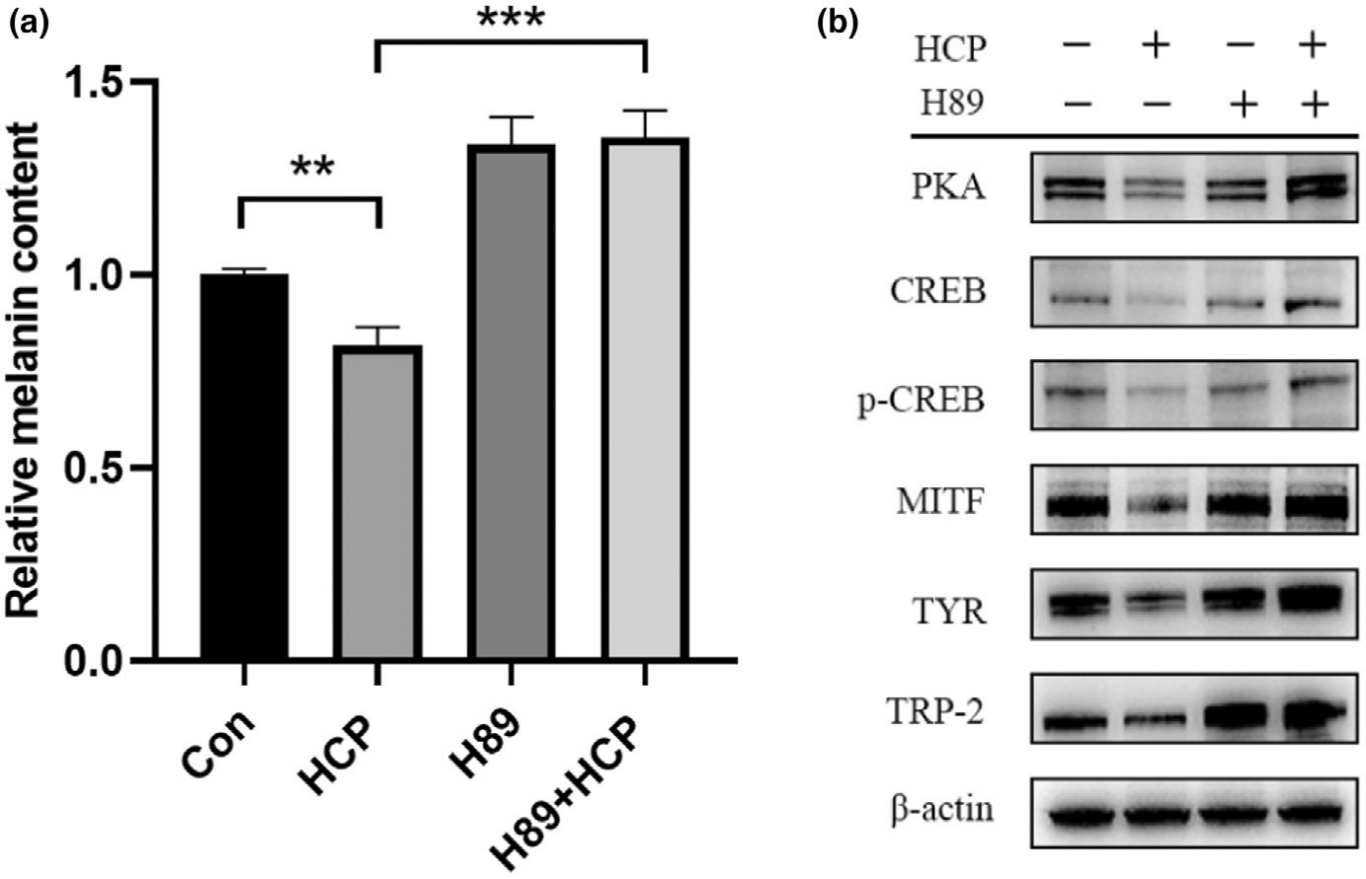
Recovery effect of co‐treatment with PKA specific inhibitors and HCP on cellular melanin content and expression of melanogenesis‐related proteins in B16F10 cells. Cells were treated with (or without) 10 μM PKA inhibitor (H89) for 1 h and further incubated with 50 μg/mL HCP for 24 h. (a) The cellular melanin content was evaluated. Data are displayed as mean ± SD (*n* = 3). (b) The expression of melanogenesis‐related proteins was analysed by western blotting. Equal protein loading was confirmed through comparison with β‐Actin expression. Data are displayed as mean ± SD (*n* = 3). *p < 0.05, **p < 0.01, ***p < 0.001. Con, Control.

To elucidate the mechanism by which HCP inhibits melanogenesis, we investigated the effect of HCP on the PKA/CREB signalling pathway. The results in Figure 2 show that, after treatment with HCP, the protein expression levels of PKA, CREB, and p‐CREB decreased. Subsequently, we conducted further experiments to validate whether HCP regulates melanin production through the PKA/CREB signalling pathway. B16F10 cells treated with HCP were exposed to a specific PKA inhibitor (H89), and the suppresion of melanin production was reversed by the PKA inhibitor (Figure 4a). The expression levels of PKA, CREB, and p‐CREB increased after treatment with HCP when the PKA inhibitor was added. Additionally, the downstream protein expression levels of MITF, TYR, and TRP‐2 also increased (Figure 4b). These results indicate that HCP can suppress melanin expression by inhibiting the PKA/CREB pathway.

### HCP inhibits melanogenesis through the MEK/ERK pathway

**FIGURE 5:**
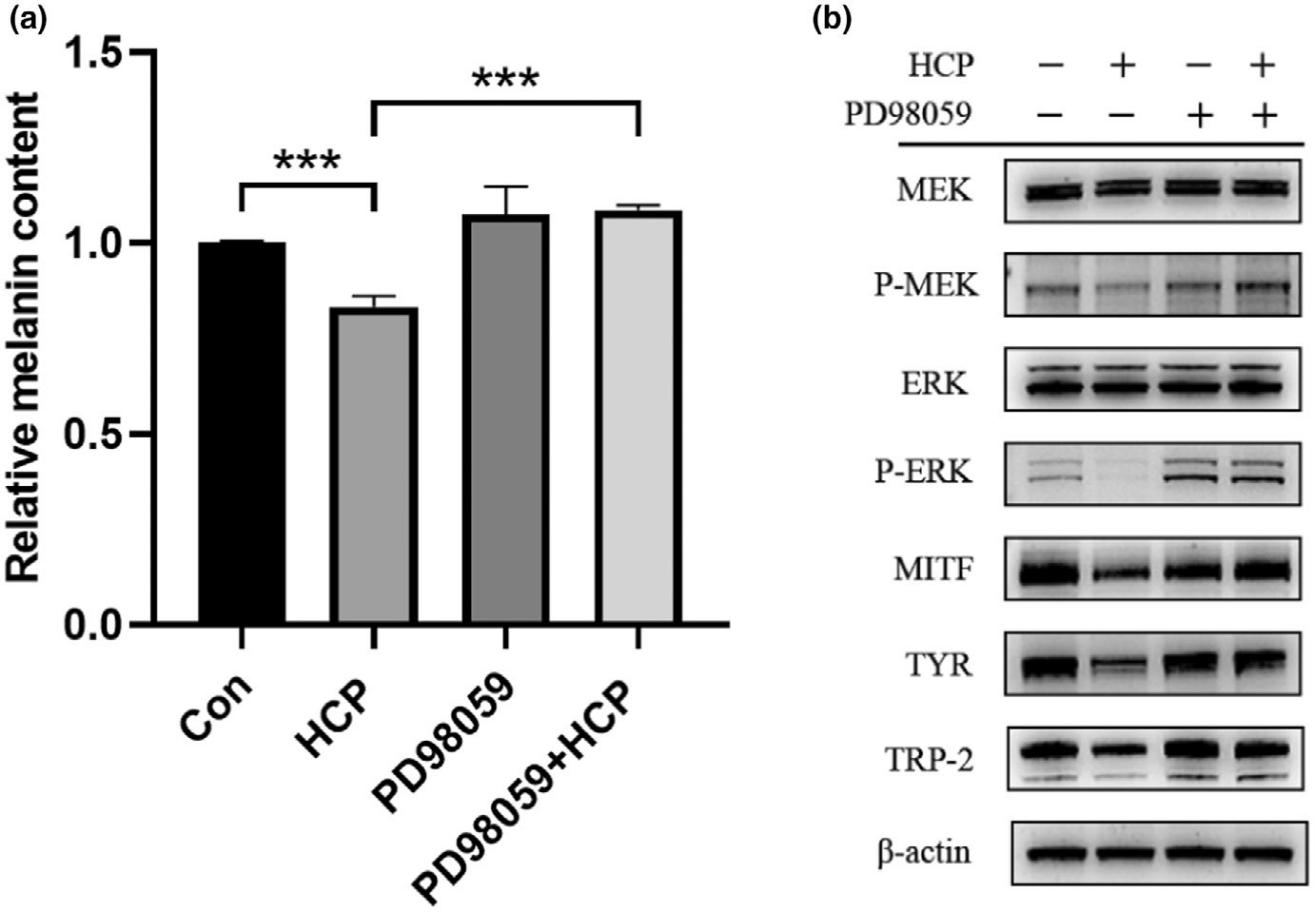
Recovery effect of co‐treatment with MEK specific inhibitors and HCP on cellular melanin content and melanogenesis‐related proteins expression in B16F10 cells. Cells were treated with (or without) 10 μM MEK inhibitor (PD98059) for 1 h and further incubated with 50 μg/mL HCP for 24 h. (a) The cellular melanin content was evaluated. Data are displayed as mean ± SD (*n* = 3). (b) The expression of melanogenesis‐related proteins was analysed by western blotting. Equal protein loading was confirmed through comparison with β‐Actin expression. Data are displayed as mean ± SD (*n* = 3). *p < 0.05, **p < 0.01, ***p < 0.001. Con, Control.

We investigated the effect of HCP on several MEK/ERK signalling pathway proteins. As shown in Figure 2, MEK and ERK expression did not change significantly after addition of HCP, while p‐MEK and p‐ERK protein expression was downregulated. Additionally, we investigated the changes in melanin inhibition with and without PD98059 (MEK inhibitor). Compared with the HCP‐treated group, the group treated with HCP + PD98059 showed a significant increase in melanin production (Figure 5a). Furthermore, as depicted in Figure 5b, exposing HCP‐treated B16F10 cells to the specific inhibitor PD98059 resulted in elevated levels of p‐MEK and p‐ERK along with increased protein expression levels of downstream MITF, TYR, and TRP‐2. These results indicate that HCP suppresses melanin expression by inhibiting the MEK/ERK signalling pathway.

### The effect of HCP on pigmentation in zebrafish

**FIGURE 6:**
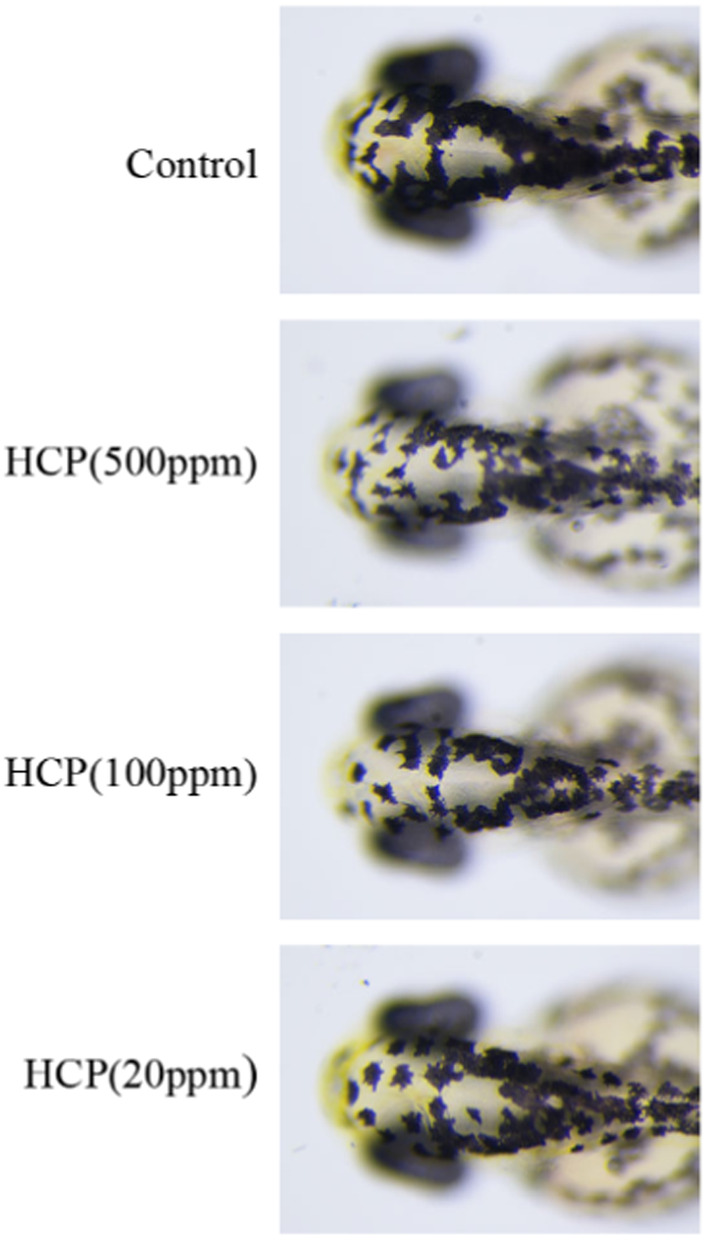
Effect of HCP on pigmentation in zebrafish. Representative photographs of zebrafish. Zebrafish embryos were treated with HCP from 35 to 60 h. The effects on zebrafish pigmentation were observed under the stereomicroscope.

Melanin pigmentation on the surface of zebrafish could be observed simply, without the need for complex experimental procedures [25]. In this study, arbutin (3000 ppm) was used as a positive control. As shown in Figure 6, HCP at 500 ppm, 100 ppm, and 20 ppm reduced pigmentation in zebrafish, with inhibition rates of 32%, 13%, and 11%, respectively.

### Evaluation of the whitening effect of HCP in a 3D melanin skin model

**FIGURE 7:**
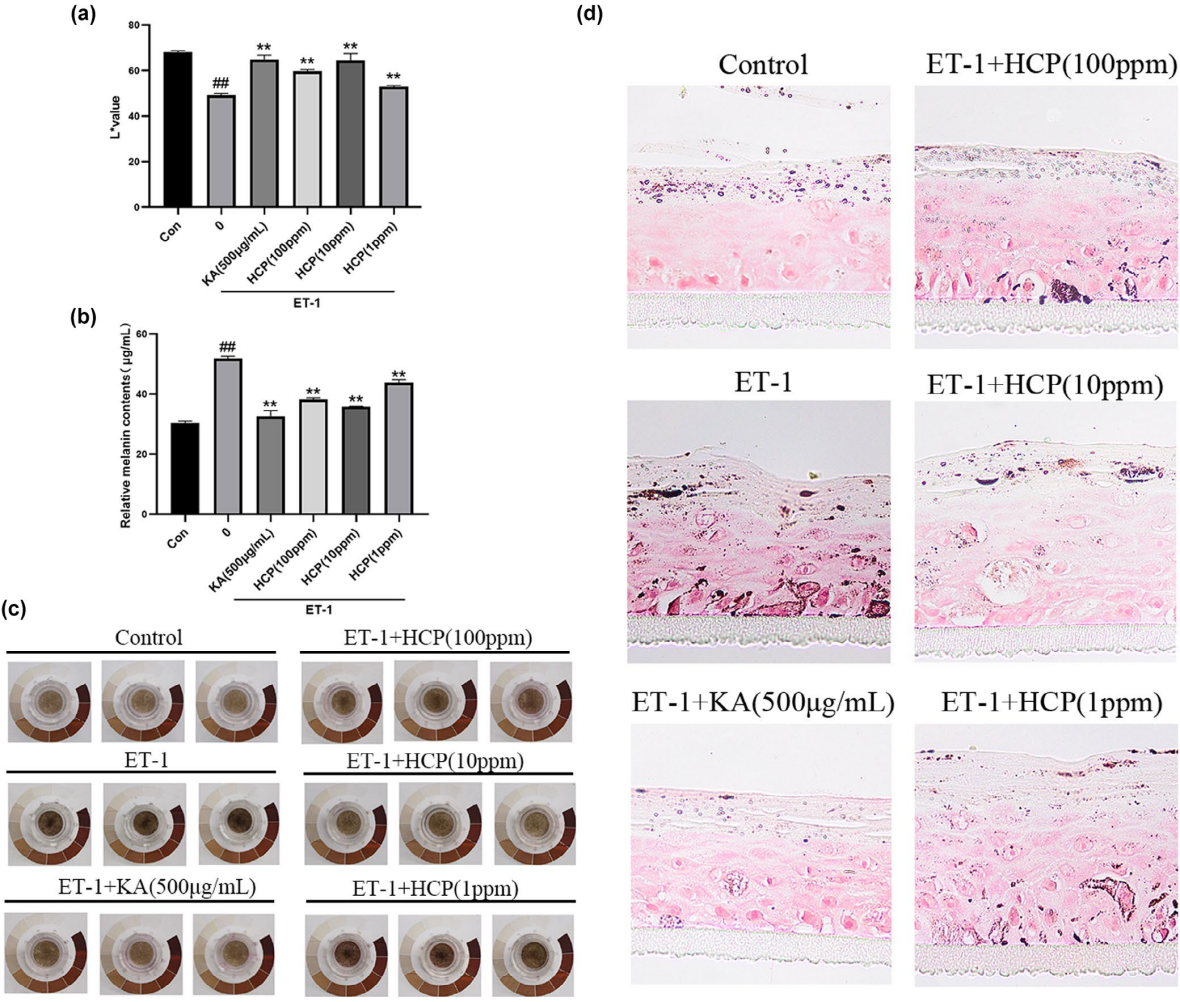
Effect of HCP in a 3D Melanin Skin Model. (a) Apparent brightness (L* value) (b) melanin content (c) apparent chroma (d) melanin distribution.

The results based on the 3D melanin skin model are shown in Figure 7. Compared with the control group, at concentrations of 100 ppm, 10 ppm, and 1 ppm, there was a significant whitening effect in terms of apparent chroma (c); a significant improvement in apparent brightness with improvement rates of 21.41%, 31.04%, and 7.52%, respectively (a); melanin inhibition rates of 26.26%, 30.90%, and 15.55% respectively (b); and a significant reduction in melanin granules with inhibition rates of 58.43%, 72.85%, and 40.45% (d). This suggests that HCP can achieve a whitening effect by improving apparent chroma, enhancing apparent brightness, and inhibiting melanin synthesis.

## DISCUSSION

Pearl and nacre, in addition to being used as jewellery, serve as ingredients in cosmetics and pharmaceutical products. Recent advancements have shed light on the material basis and mechanisms of the skin‐lightening effect of pearl powder. Studies indicate that pearl powder and pearl extracts can counteract α‐MSH and ET‐1, inhibiting proteins crucial for melanin synthesis and transport. This inhibitory effect yields a reduction in melanin production and contributes to skin‐whitening [19, 26]. Shan Yang found that HCP antagonizes the effects induced by α‐MSH‐ and ET‐1 on MNT‐1 human melanoma cells; moreover, it promotes melanin transfer to keratinocytes, inhibits TYR activity, and reduces the levels of proteins associated with melanogenesis [20]. Previous studies have indicated that HCP can abrogate the stimulating effects of melanogens; however, the signalling pathways involved in melanogenesis inhibition remain unclear. Herein, instead of using melanogenic induction, we directly added HCP to investigate its effects on melanin synthesis.

We first found that HCP had no cytotoxic effect on B16F10 in the range of 0–100 μg/mL and on PIG1 cells in the range of 0–50 μg/mL, using in vitro cytotoxicity assays. On this basis, the effects of various concentrations of HCP on melanin production in B16F10 cells and PIG1 cells were investigated. HCP was found to decrease melanin production in a concentration‐dependent manner. These results suggest that HCP may be utilized in cosmetics as a natural active ingredient against pigmentation.

To further understand the melanin‐inhibiting effect of HCP, we first examined the effect of HCP on the expression of melanogenesis‐related proteins in B16F10 cells, and found that MITF, TYR, and TRP2 were significantly decreased after HCP treatment. HCP inhibited MITF and TYR expression in PIG1 cells upon α‐MSH stimulation. These results suggest that HCP inhibits melanogenesis by downregulating MITF and TYR in melanocytes. It has been shown that inhibition of MITF and TYR is an important strategy for skin whitening, making the assessment of MITF expression a useful method for identifying skin‐whitening substances [27]. Thus, our data suggest that HCP exerts skin‐lightening effects. MITF plays a central role in melanin synthesis, melanosome development, and transport [28]. MITF expression is regulated by multiple signalling pathways, such as PKA/CREB, MAPK, and WNT [29]. PKA is involved in CREB phosphorylation, which can induce MITF transcription [30, 31]. We speculate that the observed effects of HCP are mediated by signalling pathways that inhibit MITF expression through activation of PKA and CREB phosphorylation. As expected, our data clearly show that HCP inhibited the expression of PKA and CREB and their phosphorylation in B16F10 cells. Furthermore, or findings show that the specific PKA inhibitor H89 partially restored the melanin‐inhibiting effect of HCP as well as the expression of the above proteins, indicating that the melanin inhibition effect of HCP is mediated through inhibition of the PKA/CREB pathway.

MAPK signalling pathways include p38, ERK, and JNK, which regulate melanin synthesis, and MITF expression is MAPK‐regulated [32, 33, 34]. Phosphorylation of ERK and p38 has been found to increase MITF expression and promote melanogenesis, while phosphorylation of JNK inhibits melanogenesis [35, 36]. Our results showed that HCP inhibited MEK/ERK phosphorylation and, ultimately, MITF, TYR, and TYR‐related protein expression. However, HCP had no effect on p38 and JNK phosphorylation (not shown). We found that the MEK/ERK inhibitor PD98059 significantly restored melanin synthesis as well as ERK signalling pathway protein expression. These results suggest that the melanin‐inhibiting effect of HCP is mediated by suppression of the MEK/ERK pathway.

In previous studies, we revealed that HCP inhibits tyrosine activity and melanin synthesis in B16F10 cells following ET‐1 induction [19]. Here, we conducted a more in‐depth investigation by evaluating an ET‐1‐stimulated human 3D skin model for changes in the apparent chroma, apparent brightness (L* value), melanin content, and melanin distribution post‐HCP treatment. The results verified that HCP exerts a skin‐whitening effect. Similarly, in the zebrafish model, HCP also demonstrated remarkable skin‐lightening efficacy.

**FIGURE 8:**
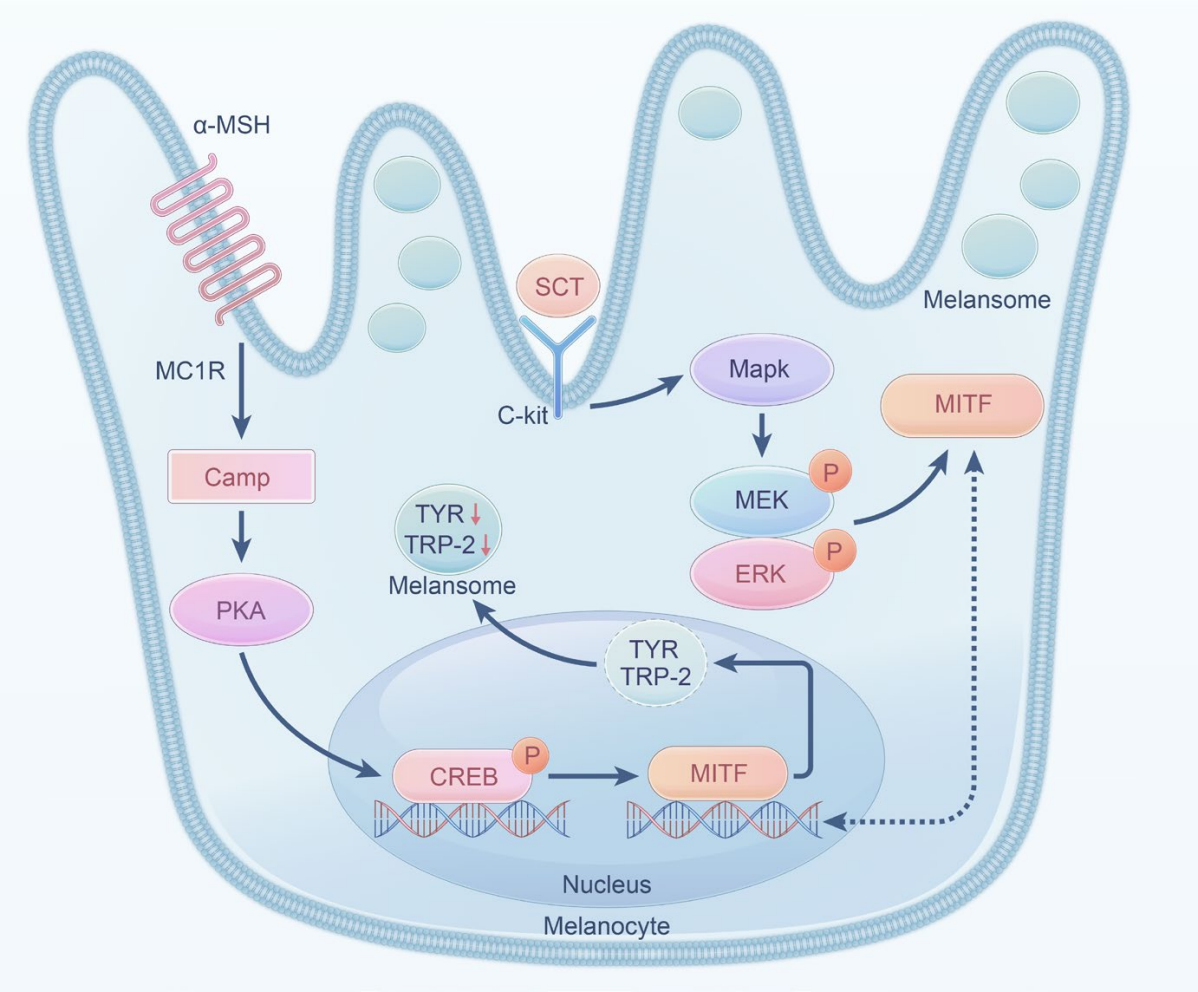
Schematic model of melanogenic modulation by PKA/CREB, MEK/ERK signalling treated with HCP.

In conclusion, our findings suggest that HCP possesses an anti‐melanogenic properties. HCP affects MITF expression and inhibits melanogenesis by regulating the PKA/CREB, MEK/ERK pathways (Figure 8). In human 3D skin model and zebrafish model experiments, HCP inhibited melanin synthesis, yielding skin‐whitening effects. Considering the low cytotoxicity observed in our study, HCP emerges as a potentially safe efficient whitening skin whitening agent.

## AUTHOR CONTRIBUTIONS

Yaqi Zhang and Sisi Wang performed experiments. Yaqi Zhang prepared the manuscript. Yaqi Zhang designed the study and revised the paper. All authors read and reviewed the manuscript.

## FUNDING INFORMATION

N/A.

## CONFLICT OF INTEREST STATEMENT

The authors declare no conflicts of interest.

## CONSENT

Informed consent was obtained from all subjects involved in the study.

## PUBLISHERS NOTE

The statements, opinions and data contained in all publications are solely those of the individual author(s) and contributor(s) and not of MDPI and/or the editor(s). MDPI and/or the editor(s) disclaim responsibility for any injury to people or property resulting from any ideas, methods, instructions or products referred to in the content.

## Data Availability

Not applicable.
